# Primary Laryngeal Lymphoma: A Case Report of a Rare Entity

**DOI:** 10.7759/cureus.72626

**Published:** 2024-10-29

**Authors:** Shravanthi Mantra Prithviraj, Shyam Sudhakar Sudarsan, Manoj Kumar L

**Affiliations:** 1 Otorhinolaryngology, Saveetha Institute of Medical and Technical Sciences, Chennai, IND; 2 Otorhinolaryngology, Saveetha institute of medical and technical sciences, Chennai, IND

**Keywords:** chemotherapy, chop regimen, diffuse large b-cell lymphoma, non-hodgkin lymphoma, primary laryngeal lymphoma, supraglottic masses

## Abstract

Primary laryngeal lymphoma is a rare form of non-Hodgkin lymphoma (NHL), with fewer than 100 cases reported worldwide. Although head and neck cancers are the second most common extranodal sites for NHL, the larynx, particularly the supraglottic region, is rarely involved. We present a case that underscores the importance of distinguishing primary laryngeal lymphoma from secondary involvement to ensure the appropriate management of the condition.

A 53-year-old male presented with significant weight loss, hoarseness, and progressive dysphagia. He also reported an intermittent history of fever and night sweats. Physical examination revealed bilateral level IIa and III cervical lymphadenopathy and flexible laryngoscopy showed edema and ulceration in the supraglottic region. A contrast-enhanced CT scan from the skull base to the thorax revealed a fairly well-defined, mildly heterogeneous enhancing lesion involving the suprahyoid and glottic regions, measuring 2.3 x 2.1 x 5 cm (AP x TR x CC). Biopsy confirmed diffuse large B-cell lymphoma (DLBCL), with immunohistochemistry positive for CD45, CD20, and BCL6. The patient received eight cycles of the CHOP (doxorubicin, cyclophosphamide, prednisone, and vincristine) chemotherapy regimen, achieving complete clinical remission with no recurrence noted at the two-year follow-up.

DLBCL of the larynx is rare but should be considered in cases of supraglottic masses due to its symptom overlap with other laryngeal conditions. Diagnosis is confirmed through histopathology and immunohistochemistry, and treatment typically involves chemotherapy, with CHOP being the most effective regimen. Early recognition is crucial for optimal treatment of this uncommon disease. Further research is needed to refine treatment protocols.

## Introduction

Fewer than 100 cases of primary laryngeal lymphoma have been reported worldwide, making it an extremely rare condition. Although the head and neck region is the second most common extranodal site for non-Hodgkin lymphoma (NHL), the larynx itself is rarely affected [1]. Primary laryngeal lymphomas are typically NHLs and are most commonly found in the supraglottic region, which is rich in follicular lymphoid tissue. It is crucial to differentiate primary laryngeal lymphomas from secondary involvement due to systemic or leukemic conditions to facilitate accurate diagnosis and appropriate management [2].

Laryngeal involvement by hematopoietic neoplasms, such as NHL and extramedullary plasmacytoma, predominantly occurs in older males and often presents with a short history of hoarseness, dysphagia, and other symptoms such as snoring or progressive respiratory difficulty [1,3]. The supraglottic structures, particularly the epiglottis and aryepiglottic folds, are the most frequently affected sites, with lesions typically presenting as non-ulcerated, polypoid growths. Early laryngeal visualization and timely intervention are essential for improving outcomes in these patients, as the rarity and nonspecific presentation of the condition often lead to delayed diagnosis [4].

## Case presentation

A 53-year-old man presented with concerns of mild difficulty swallowing solid foods and a persistent foreign body sensation in his throat for the past five months. In addition to dysphagia, he reported progressively worsening hoarseness over the last three months. Initially mild, the hoarseness had gradually intensified, leading to significant changes in voice quality. These symptoms were accompanied by an unexplained weight loss of approximately 6 kg over two months, which was concerning. He also reported an intermittent history of fever and night sweats. The patient denied any episodes of hemoptysis, difficulty breathing, or stridor. He also had no history of smoking, reported minimal alcohol consumption, and had no exposure to environmental or occupational risk factors such as dust or chemicals. The history of multiple fine needle aspiration cytology (FNAC) done outside was inconclusive.

On general physical examination, the patient appeared moderately built but mildly cachectic. His vital signs were stable, with a blood pressure of 130/85 mmHg, heart rate of 76 beats per minute, and oxygen saturation of 98% on room air. A comprehensive ENT examination revealed normal findings in the ears and nose. The oral cavity examination was unremarkable, with no deviation of the uvula, impaired mobility of the soft palate, or medialization of the pillars. Neck examination showed bilateral level IIa and III cervical lymphadenopathy, with 2x2 cm, firm, and non-tender lymph nodes. The thyroid gland was normal on palpation, and laryngeal crepitus was present, with no laryngeal tenderness.

**Figure 1:**
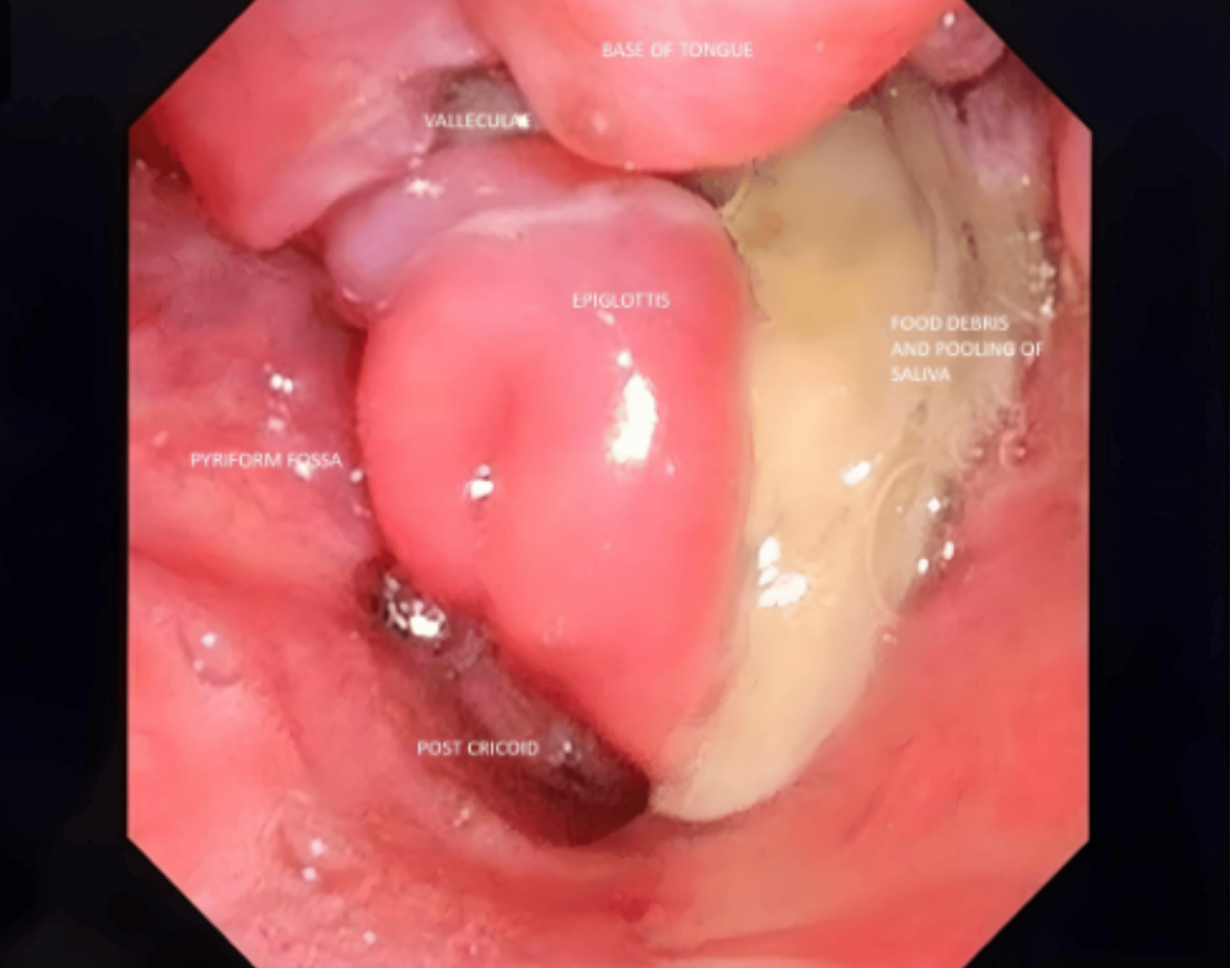
Fibreoptic laryngoscopy The image shows edema of the aryepiglottic fold and epiglottis with areas of irregular ulcers, deviation of the epiglottis, and obstruction of the upper airway. A large slough-covered area with food debris was noted in the left pyriform sinus with a pooling of saliva

Flexible laryngoscopy revealed edema of the aryepiglottic fold and epiglottis, with areas of irregular ulceration, deviation of the epiglottis, and upper airway obstruction. A large slough-covered area with food debris was observed in the left pyriform sinus, along with saliva pooling. Bilateral vocal cords were not fully visualized and exhibited impaired mobility (Figure 1).

**Figure 2:**
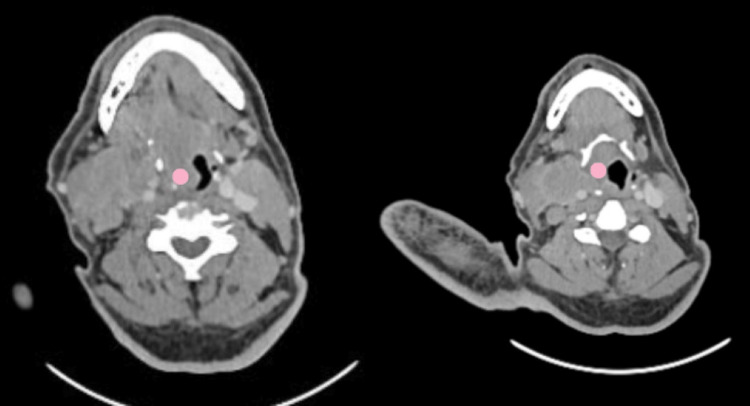
Contrast-enhanced CT scan from the skull base to the thorax The images reveal a fairly well-defined, mildly heterogeneous enhancing lesion involving the suprahyoid and glottic regions, measuring 2.3 x 2.1 x 5 cm (AP x TR x CC). On post-contrast imaging, the lesion showed heterogeneous enhancement and was observed to extend superiorly into the right laryngeal vestibule and vestibular fold. Inferiorly, the lesion was seen invading and displacing the right true vocal cord medially and the cricovocal membrane anteriorly, with invasion into the preglottic space. There was no evidence of involvement of the prevertebral space or erosion of the vertebrae posteriorly. Medially, the lesion crossed the midline, and there was obliteration of the bilateral valleculae, more pronounced on the right side than on the left CT: computed tomography

A contrast-enhanced CT scan from the skull base to the thorax revealed a fairly well-defined, mildly heterogeneous enhancing lesion involving the suprahyoid and glottic regions, measuring 2.3 x 2.1 x 5 cm (AP x TR x CC). On post-contrast imaging, the lesion showed heterogeneous enhancement and was observed to extend superiorly into the right laryngeal vestibule and vestibular fold. Inferiorly, the lesion was seen invading and displacing the right true vocal cord medially and the cricovocal membrane anteriorly, with invasion into the pre-epiglottic space. There was no evidence of involvement of the prevertebral space or erosion of the vertebrae posteriorly. Medially, the lesion crossed the midline, and there was obliteration of the bilateral valleculae, more pronounced on the right side than on the left (Figure 2).

**Figure 3:**
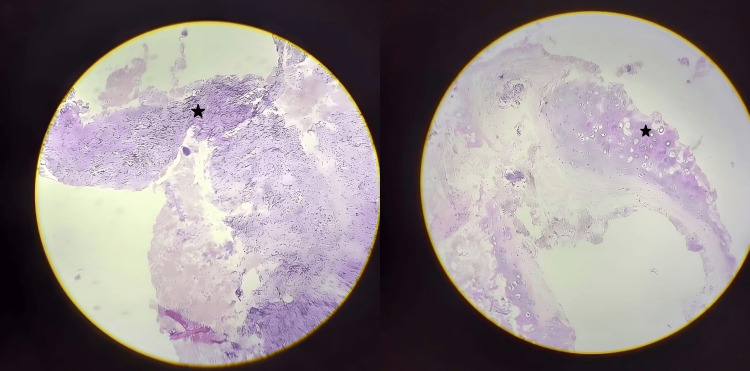
Histopathology sections The images reveal multiple tissue fragments consisting of irregular sheets of large cells with scant to moderate eosinophilic cytoplasm and moderately pleomorphic vesicular nuclei. Many of these cells showed prominent nucleoli and an increased number of mitotic figures, confirming the presence of a lymphoproliferative disorder/poorly differentiated carcinoma (*)

There was no evidence of distant spread, clinically classifying the disease as Ann Arbor Stage IIE. Further management included an elective tracheostomy (anticipating airway compromise), followed by a direct laryngoscopy-guided biopsy of the supraglottic lesion under general anesthesia. Histopathology sections revealed multiple tissue fragments consisting of irregular sheets of large cells with scant to moderate eosinophilic cytoplasm and moderately pleomorphic vesicular nuclei. Many of these cells showed prominent nucleoli and an increased number of mitotic figures, confirming the presence of a lymphoproliferative disorder/poorly differentiated carcinoma (Figure 3).

**Figure 4:**
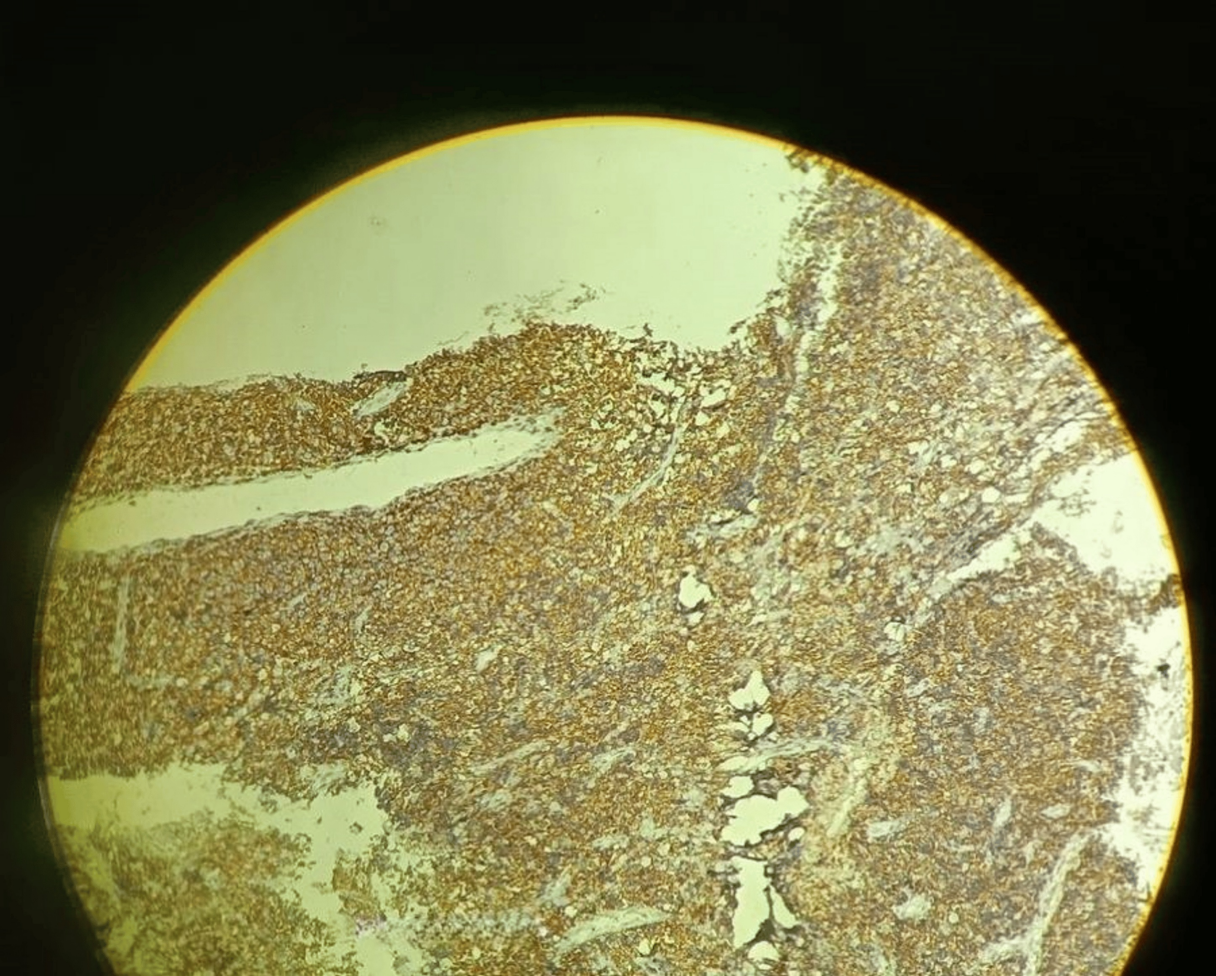
Immunohistochemical staining The staining showed strong membranous positivity for CD45 in 100% of the cells. Diffuse large B-cell lymphoma had been indicated by the tumor cells' positive results for CD20 and BCL6 as well as negative results for CK and CD3

Immunohistochemical staining showed strong membranous positivity for CD45 in 100% of the cells. Diffuse large B-cell lymphoma (DLBCL) was indicated by the tumor cells' positive results for CD20 and BCL6, as well as negative results for CK and CD3 (Figure 4).

Staging with contrast-enhanced CT of the neck and a PET-CT scan demonstrated Lugano classification Stage II (limited), which indicated localized disease confined to the laryngeal region with involvement of cervical lymph nodes at levels II and III. However, there was no evidence of distant spread, classifying the disease as Ann Arbor Stage IIE.

**Figure 5:**
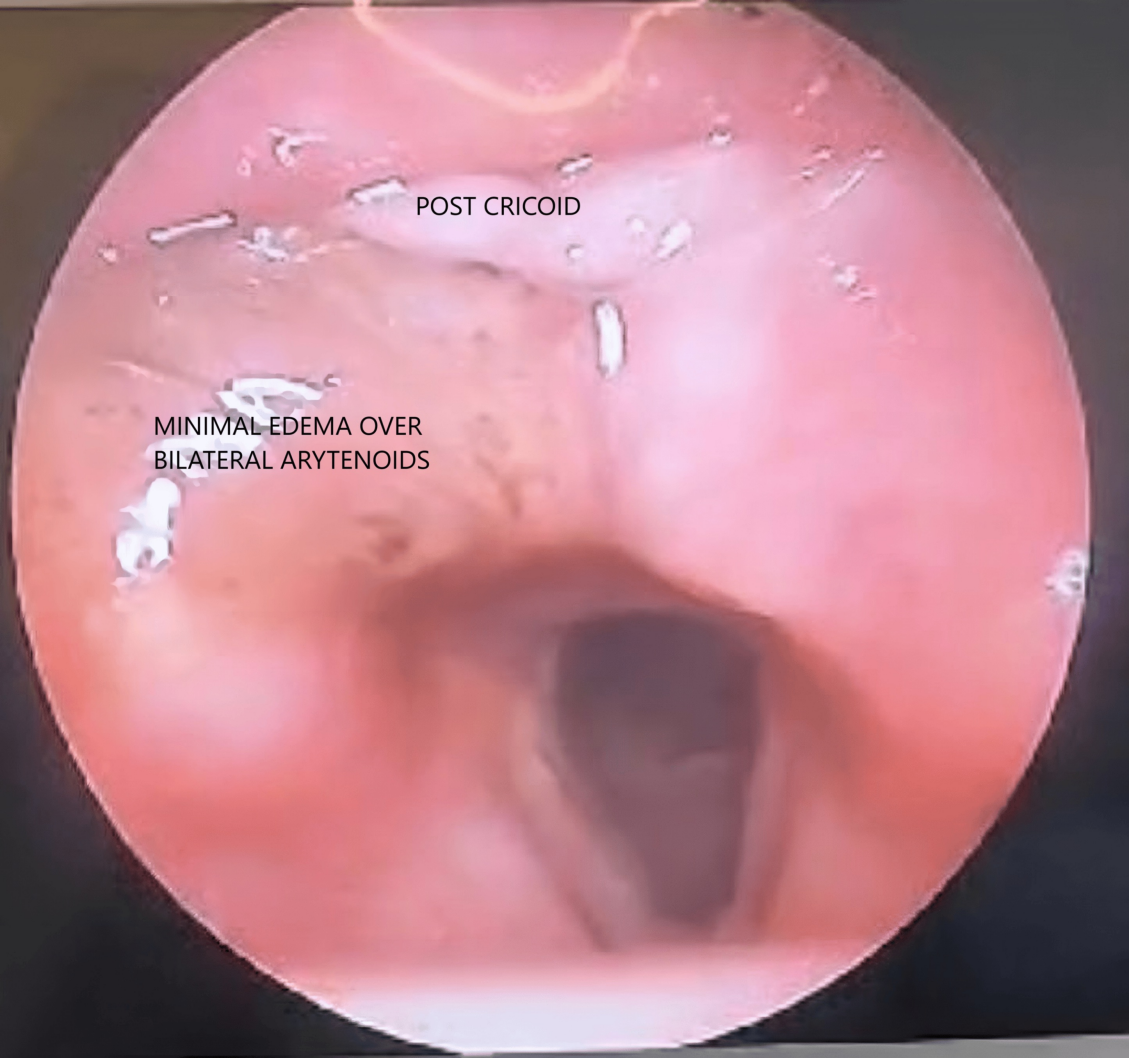
Video laryngoscopy at the two year follow-up The image shows a clear visualization of the supraglottic, glottic, and subglottic regions. The mucosal surface appeared smooth and intact, with no visible signs of ulceration, granulation tissue, or erythema. Vocal cords were fully mobile bilaterally, with symmetrical movement during phonation. No residual mass or irregularity was noted. The surrounding structures, including the arytenoids and epiglottis, appear normal, with minimal edema. Airway patency was intact, and no obstructive lesions were observed

As the patient could not afford the R-CHOP regimen, he underwent eight cycles of chemotherapy using the CHOP regimen (vincristine, prednisone, cyclophosphamide, and doxorubicin), which gave on par or better overall response rate. Upon completion of chemotherapy, a video-laryngoscopy showed no clinically apparent soft tissue lesions and minimal pooling of saliva (Figure 5). The patient was subsequently advised to undergo speech and swallowing therapy and was rehabilitated. At the two-year follow-up, following multiple hospital visits, no new lesions or residual disease were noted, and the patient's condition was observed to have symptomatically improved.

## Discussion

Primary DLBCL of the larynx is a rare form of NHL but should be considered in cases of supraglottic masses due to its unique clinical presentation and management compared to other laryngeal malignancies such as squamous cell carcinoma, adenocarcinoma, MALToma, extramedullary plasmacytoma, and sarcomas [1]. The more common extranodal sites for NHL include the paranasal sinuses, salivary glands, and thyroid glands, making primary involvement of the larynx an unusual occurrence. When it does occur, DLBCL predominantly affects the supraglottic area due to the presence of lymphoid tissue in this region [2].

Symptoms of laryngeal lymphoma are often nonspecific and may overlap with other benign or malignant conditions. Typical symptoms include dysphonia, dysphagia, hoarseness, and dyspnea, while cervical lymphadenopathy, cough, and, occasionally, systemic symptoms such as weight loss and fever may also be present [3]. Due to its nonspecific presentation, laryngeal lymphoma is often misdiagnosed as other conditions such as cystic lesions, recurrent respiratory papillomatosis, or laryngeal stenosis. Some patients are even mistakenly diagnosed with asthma or other conditions associated with wheezing. This underscores the importance of maintaining a high index of suspicion when evaluating persistent supraglottic masses, especially when common treatments fail to provide relief [4-7].

Diagnosis of primary laryngeal DLBCL requires a combination of clinical, radiological, and histopathological evaluations. FDG-PET/CT scans are recommended by the Lugano classification as the gold standard for staging DLBCL, as they are more sensitive in detecting bone marrow involvement than traditional bone marrow biopsy techniques [7]. When FDG-PET shows focal bone marrow uptake, the likelihood of bone marrow infiltration is high, highlighting the importance of PET imaging in staging and management. A biopsy is essential to confirm the diagnosis and guide the treatment plan. Chemotherapy is the primary treatment for DLBCL, using regimens similar to those for Burkitt lymphoma. The addition of immunotherapy, particularly rituximab in combination with the CHOP regimen, has significantly improved patient outcomes [8-11]. Coiffier et al. have demonstrated that rituximab not only increases complete response rates but also reduces relapse and treatment failure, leading to improved overall survival compared to CHOP alone [9].

Surgery is generally not indicated but may be necessary in cases of extensive airway obstruction or emergencies to secure the airway. Most cases are managed non-surgically with chemotherapy and radiotherapy, as laryngeal lymphomas typically remain localized for extended periods without significant progression. Radiation therapy is effective for local disease control, while systemic chemotherapy addresses potential dissemination [12,13]. Overall, treatment strategies for primary laryngeal lymphoma align with standard NHL management, utilizing a multimodal approach that includes chemotherapy, radiotherapy, and immunotherapy [14]. Given the rarity of this condition, a tailored treatment plan is required, based on the patient's overall health, tumor stage, and airway status. Further research and case studies are needed to refine treatment protocols and improve long-term outcomes for individuals with this uncommon presentation of DLBCL.

## Conclusions

Primary DLBCL of the larynx is a rare condition that should be considered in the differential diagnosis of supraglottic neck masses. Given its atypical presentation, early detection and accurate diagnosis are crucial for effective treatment and improved outcomes. Due to the rarity of primary laryngeal lymphoma, standardized treatment protocols are not well-defined. However, it should be managed according to current NHL guidelines, as it represents an unusual presentation of NHL rather than a separate disease entity.
